# Motives of Dutch persons aged 50 years and older to accept vaccination: a qualitative study

**DOI:** 10.1186/s12889-015-1825-z

**Published:** 2015-05-16

**Authors:** Renske Eilers, Paul F. M. Krabbe, Hester E. de Melker

**Affiliations:** University of Groningen, University Medical Center Groningen, Department of Epidemiology, P.O. Box 30.001, 9700 RB Groningen, The Netherlands; Center for Infectious Disease Control, National Institute for Public Health and the Environment (RIVM), P.O. Box 1, 3720 BA Bilthoven, The Netherlands

**Keywords:** Vaccination, Older adults, Decision-making, Susceptibility, Tailored vaccination, Focus groups

## Abstract

**Background:**

Elderly in several European countries are currently being vaccinated against influenza and pneumococcal disease, and various reasons have been put forward to expand these programs. To successfully immunize the older adult population, however, it is crucial for the target group to accept such interventions. This study aims to elucidate the motives of Dutch persons aged ≥50 years for accepting vaccination.

**Methods:**

Thirteen focus groups were composed with persons aged 50 years and older. A semi-structured topic list with open-ended questions was used to guide the focus groups. The transcripts were analyzed according the principles of thematic survey. By an inductive process, the main themes and related subthemes were extracted from the responses.

**Results:**

Eight themes were found to play an important role in accepting vaccination: healthy aging; usefulness of vaccination in older age; risk of getting an infectious disease; vaccine characteristics; severity of the disease and its implications; the experiences of previous vaccinations; the influence of healthcare workers and other people; and the need for information.

**Conclusions:**

This qualitative study reveals that acceptance of vaccination is not based on a single argument. The most important one appears to be the risk of getting an infectious disease. In that light, vaccination campaigns may emphasize the susceptibility of older adults. It is also advisable to consider the usefulness of vaccination in older age as an overall argument. A tailored approach to offering vaccination may be considered. Further research would be needed to determine the relative importance of the factors identified in this study.

**Electronic supplementary material:**

The online version of this article (doi:10.1186/s12889-015-1825-z) contains supplementary material, which is available to authorized users.

## Background

Europe is aging; it is estimated that by 2060, 28.4 % of the population of the 27 Member States of the European Union will be 65 years or older, compared to 18.6 % in 2014 [1]. As a result of immunosenescence (the gradual deterioration of the immune system), co-morbidity, and general frailty, this population is susceptible to infectious diseases [2], resulting in higher mortality and morbidity rates than in young adults [3]. Infections may lead to irreversible frailty and thereby further dependency on long-term healthcare [4]. At the same time, community-dwelling older adults will be more socially engaged, which increases the chance of transmission of infectious diseases towards this population [5]. Apart from possible benefits to individuals in this age group, vaccination may yield social benefits such as lower overall costs of healthcare, as demonstrated by childhood vaccinations [6].

Several European countries offer elderly vaccination against influenza and in some instances against pneumococcal disease, tetanus, and diphtheria. In the Netherlands, for example, influenza vaccination is offered to everyone of 60 years or older and pneumococcal vaccine to groups at risk. However, proposals have been made to expand the Dutch immunization program for persons aged 60 years and older to include pneumococcal disease, herpes zoster, and pertussis [7].

For an immunization program to be successful and produce the most health benefits, its acceptance is crucial. It is therefore necessary to understand why the different reasons given by older persons accept or reject vaccination. So far, no qualitative study has explored these arguments for persons aged 50 years and older in the Netherlands. The aim of this study is to explore the motives to accept or refuse vaccination among community-dwelling persons aged 50 years and older in the Netherlands.

## Methods

### Participants and procedures

In total, 13 focus groups of individuals of 50 years or older (n = 80) were composed. In a focus group, a person’s view is clarified through interaction with other people, which does not occur in an interview [8]. It was decided to take 50 years as the cutoff point because of the possible biological advantages of starting vaccination earlier in life (*e.g.*, stronger immune response).

A list of foundations for the welfare of older adults, sheltered housing institutions, care homes, and residential groups across the Netherlands was compiled based on an internet search. Locations were selected from this list, whereby the geographical distribution and degree of urbanization were taken into account to ensure nationwide distribution and inclusion of individuals in both urban and rural areas. In addition, two commercial agencies were approached to recruit persons aged 50 and older. Letters were sent out to the different organizations inviting persons to participate in the study. Candidate respondents received an information letter describing the background, objectives, and procedures of the study and enclosing an informed consent form.

Those willing to take part returned the form containing their personal information. The participants ranged from 52 to 92 years of age and were classified as living independently (n = 31), in a residential group (n = 37), in a care home (n = 2), or in sheltered housing (n = 10) (Table 1).

**Table 1:** An overview of the age, sex and domestic situation per focus group

Focus group	Nr. of participants	Proportion male: female	Nr of pers. 50 a.o.^a^	Nr of pers. 60 a.o.^a^	Nr of pers. 70 a.o.^a^	Nr of pers. 80 a.o.^a^	Domestic situation
1	9	0:9	3	4	0	2	Living independent
2	7	1:6	5	2	0	0	Living independent
3	8	2:6	0	2	3	3	Residential group
4	7	0:7	1	3	3	0	Residential group
5	7	3:4	0	5	2	0	Residential group
6	9	1:8	0	1	7	1	Residential group
7	6	2:6	0	0	5	1	Residential group
8	3	1:2	0	1	0	2	Sheltered housing
9	7	1:6	0	0	4	3	Sheltered housing
10	5	1:4	0	4	1	0	Living independent
11	7	1:6	0	6	1	0	Living independent
12	3	2:1	0	1	0	2	Living independent
13	2	0:2	0	0	0	2	Care home
Total	80	15 male:65 female	9	29	26	16	

^a^Means number of persons aged [..] and older

### Focus groups

The focus groups were convened between January and November 2012. All 13 had the same moderator (RE), accompanied by an assistant to take notes. The duration of each session varied from 65 to 98 min. Every participant received a gift voucher of €20 after attending.

The groups were guided using a semi-structured open-ended topic list (see Additional file 1). Since the aim was to explore all opinions that arose, the questions were not based on existing formats such as the Health Belief Model, which might restrict the range of topics to be raised.

Each session started with an introduction to the research and the aim of the focus group. The purpose of the study was explained as follows to the participants: “In an aging society, the prevalence of infectious diseases will rise. Vaccination could protect older adults against several infectious diseases and promote healthy aging. In that light, it is important for us to know how you feel about vaccination and what your reasons are to either accept or reject vaccination.” In addition, the group members were asked permission to record the session.

The participants were then asked to give their thoughts on vaccination in general and to write down the pros and cons of accepting vaccination. This topic covered not only influenza vaccination but also, expansion of the current program to include vaccines for herpes zoster, pneumococcal disease, and pertussis. Their views were discussed during the session. Furthermore, the contribution of vaccination to healthy aging was discussed, drawing special attention to the role of the general practitioner.

### Analysis

All sessions were recorded with a digital voice recorder and the recordings were transcribed verbatim. The transcripts were analyzed with the software program Nvivo (QSR International) according to the principles of thematic survey [9]. Themes and subthemes were extracted by an inductive process. All of the transcripts were coded by the moderator (RE); afterwards, one was also coded by an independent researcher (IH). Coding consisted of labeling passages with concepts abstracted from this text. The results were compared, discussed, and refined until consensus was reached on the coding scheme and the labelling criteria. As the schemes of RE and IH were almost identical, consensus was achieved. By coincidence, the participants in some focus groups were predominantly 70 years and older, whereas those in other groups were predominantly between 50 and 70. Therefore, efforts were made to perform some data analysis based on age by comparing the concepts identified in the transcripts of these focus groups, referred to as older and younger participants.

## Results

**Fig. 1:**
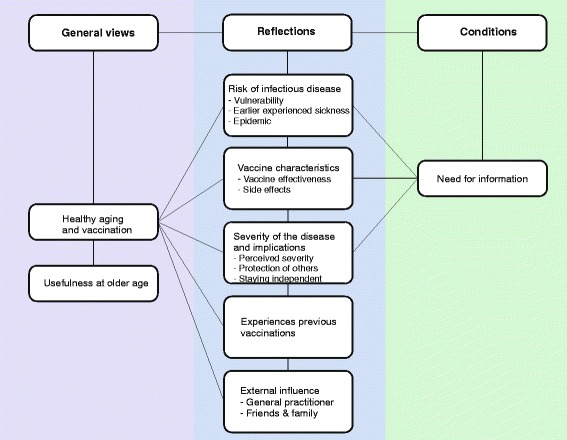
An overview of the themes and factors identified during the focus groups

The views on healthy aging and the main factors influencing the participants’ willingness to accept vaccination are discussed below and illustrated by quotes from different focus groups. The results are presented in three main topics: general views on healthy aging; reflections on whether to accept or reject vaccination; and conditions that have to be met before accepting vaccination. Each topic covers several themes that were probed during the focus groups. The general views refer to ‘healthy aging’ and ‘usefulness of vaccination in older age’. The reflections were identified as ‘the risk of getting an infectious disease’, ‘vaccine characteristics’, ‘severity of the disease’, ‘experience of previous vaccinations’, and ‘the influence of the healthcare worker and other people’. Last, ‘the need for information’ was identified as a condition (Fig. 1).

### A. General views

#### A.1 Healthy aging

There was consensus that aging is something one undergoes but that how one ages can be influenced, for example by taking care of one's body. Healthy living was specified as eating healthy food, exercising, getting enough sleep, and having no stress. *“Well, one can’t do more than lead a healthy life. Eat regular meals, and the rest comes naturally, one can’t control that. Everything else just comes by itself*.” (focus group 9, female, sheltered housing) It was acknowledged that ailments come with aging; the crux is coping with them: *“As for aging gracefully, everyone gets sick now and then, life has its ups and downs, just accept it. You will suffer setbacks, so look on the bright side.”* (focus group 7, female, residential group)

They did, however, distinguish between physical and mental dimensions, whereby mental problems (such as Alzheimer’s) were perceived as more severe. As one woman said, *“Because to me, the worst thing that can happen to you is … with your mind*.” (focus group 12, female, independent)

The participants defined healthy aging as remaining independent and self-reliant. It was important for them to actively take part in society, engage in social contacts, and have a meaningful life. There was no consensus on whether vaccination could contribute to healthy aging. So even though they spoke about preventing disease by healthy living, not all considered vaccination as a part of this lifestyle: *“Well, but that is unnatural prevention.”* (focus group 1, female, independent) To some, accepting a vaccine was part of taking responsibility for your health and doing the best you can: *“Anyway, even if it does not help, at least you tried and that is encouraging.”* (focus group 8, male, residential group)

To others, vaccination was part of healthy aging. Some took it for granted and made a habit of accepting the influenza vaccine. Others felt that ‘it doesn’t hurt to try’, which coincides with the saying ‘prevention is better than cure’ mentioned by a few participants. They just wanted to stay healthy and prevent disease: *“I am in favour because it is part of overall prevention. Prevention is better than cure.”* (focus group 10, male, independent)

#### A.2 Usefulness of vaccination in older age

One important aspect that was brought up was the usefulness of vaccination at an older age. This was related to the fact that life is finite. Some participants felt you should let nature run its course instead of trying to prevent the inevitable: *“You have to die of something.”* (focus group 1, female, independent) Especially the older participants felt that prolonging life is not always the right choice.

Another concern was whether they would feel they had lived life to the fullest: *“Well yes, but if you feel your life is not yet complete, that there are still things you need to do for yourself, then I think it is alright to try to stretch it with an injection of something or other.”* (focus group 3, female, residential group) Some participants had doubts about prolonging life without adding any quality to it. In addition, death was sometimes seen as deliverance, especially when somebody is already suffering. These participants also spoke of pneumonia being the old man’s friend: *“And it makes a difference how you age. And let’s face it, there comes a time when death can be a blessing.”* (focus group, female, independent)

A different issue that was raised is wanting to age without any interventions (such as vaccines). This is related to the notion that ailments go hand in hand with age. To some participants, the perspective of having more vaccines available in the future was indicative of a medicalization of aging: *“Just let us grow old in an ordinary way. And we don’t necessarily all have to reach 90.”* (focus group 5, female, residential group)

### B. Reflections on whether to accept vaccination

When considering whether to accept a vaccine, people reflected on several issues. The following sections discuss factors that appear to be important when deciding whether to accept vaccination.

#### B.1 Risk of getting an infectious disease

The risk of getting an infectious disease seems to be one of the most important factors. It was not often mentioned explicitly but came up as a topic of concern, with multiple aspects as shown below.

##### B.1.1. Vulnerability

The first aspect is whether participants felt vulnerable to infectious disease. In general, they did not, even though their age in itself put them at risk. More important to them was their health status at the time of vaccination: *“What matters is your general state of health. Are you already chronically ill with one thing or another?”* (focus group 1, female, independent)

In the event of feeling healthy, the participants saw two possible actions: they would either accept vaccination in order to stay healthy, or they would reject it because they would feel healthy enough to fight the infection. This latter argument was expressed by participants who have confidence in their bodies because of their healthy lifestyle: *“I believe I’m healthy enough to deal with a possible bout of flu, and then I think it is not really necessary.”* (focus group 3, female, residential group)

In the event of feeling unhealthy, the participants mentioned two possible actions: to accept vaccination because they would feel vulnerable and be worried about their health; or to reject it because they would already be taking medication for co-morbidities and would not want the extra hassle. One participant spoke about being afraid that her body could not cope with an extra intervention due to the medications she was already taking: *“I just happen to think that I might be getting too much, these are all kinds of chemicals that your body has to deal with, and then you get something like this on top of it all.”* (focus group 9, female, sheltered housing)

##### B.1.2. Prior sickness

The second aspect is whether the participants had previous encounters with infectious disease. This includes personal experience, meaning having been ill themselves or seeing someone else get sick. Experience would have two possible effects on one’s willingness to be vaccinated. If people had been ill themselves or had seen a loved one suffering, they felt more vulnerable and were more inclined to accept immunization for this particular infectious disease: *“But I have had pneumonia two or three times already. And then, if you are given that advice, well, then you go along with it.”* (focus group 6, male, residential group)

On the other hand, participants without such experience did not consider themselves vulnerable at all and therefore wondered why they should accept the vaccine: *“Never been ill a day in my life, I never had the flu, so why should I do it now?”* (focus group 6, female, residential group)

It appeared that the younger participants tend not to feel vulnerable to infectious disease because of their healthy lifestyle, and fewer had experienced episodes of sickness.

##### B.1.3. Epidemic

In the case of an epidemic, vaccines would be more easily accepted because the infectious disease would be widespread in the population, increasing the chance of contracting it.

#### B.2 Characteristics of the vaccine

Besides personal factors, the characteristics of the vaccine were also part of the deliberation on whether to accept or reject a vaccine. These characteristics are its effectiveness and its side effects.

##### B.2.1. Vaccine effectiveness

Though acknowledging that 100 % effectiveness is difficult to achieve, the participants considered the effectiveness important. They mentioned it mainly in connection with influenza. *“Just like X said, viruses can also mutate very quickly, so you can never be assured that you won’t get the flu.”* (focus group 5, female, residential group) Those who were hesitant to get a flu shot felt it would not protect them because the vaccine only covers a few of the existing viruses. In addition, some wondered whether the immunity acquired from a vaccine is as strong as that acquired from undergoing the disease: *“And even so, if I go in for a shot with not a very strong dose, will my immune system then be activated enough? Wouldn’t I just be kidding myself?”* (focus group 2, male, independent)

In general, the minimum effectiveness for acceptance of a vaccine varied from 50 to 70 percent. This rate reflects the severity of the disease: the more severe, the more willing the participants were to accept a vaccine with a lower effectiveness. In addition, some participants did not consider prevention to be the main effect of vaccination. They said it could also make them less ill or reduce the amount of time they would be sick: *“Even if you can reduce your risk by 50 % that would already be a great improvement. See, 100 % risk-free is just hypothetical, it is just not realistic.”* (focus group 9, female, sheltered housing)

##### B.2.2. Side effects of the vaccine

Another important aspect is the possibility of side effects. In general, side effects were accepted as part of vaccination. In any event, mild side effects were never considered a problem. As one woman put it: *“Now, a red spot on your arm is no big deal, it could start hurting; well, then you just put a wet cloth around it and that takes care of that.”* (focus group 13, female, care home)

However, if the side effects would interfere with everyday life, the acceptance rate could be much lower: *“But if I would really be affected and I would have to stay in bed for three or four days because I would feel miserable, then I suppose I’d decline. I wouldn’t do anything like that.”* (focus group 8, female, sheltered housing)

Some participants doubted they would take the risk of potential severe side effects if they did not know whether they would actually get the disease. They compared vaccines with medications: *“Oh, well. Look, if you are sick and you know that it will cure you, then you do go along with it. But I mean, if you are not very sick yet and you know that you will get seriously ill from the injection, then I don’t know if I would want to take that risk.”* (focus group7, female, residential group)

In three focus groups, the participants also spoke about the content of the vaccine. Specifically, they believed that vaccines contain (poisonous) substances that diminish the functioning of the immune system. One participant expressed her feelings as follows: *“And then the dangers of it, because, yes, so there are … see, they don’t say much about it but so many poisonous substances are added to the vaccine and these are all stored up in your body, they poison your body, which means that your immunity is lowered even further.”* (focus group 4, female, residential group) There were also concerns about the impact of receiving more than one vaccine.

#### B.3 Severity of the disease and its implications

##### B.3.1. Perceived severity

Another consideration was the severity of the infectious disease that the vaccine would protect against. Participants would accept vaccination against diseases that would affect their quality of life, increase mortality, cause suffering, produce pain and discomfort, or lead to invalidity. One participant summarized it as follows: *“I think this applies to all of us. As for me, it is at the top of the list. Health risks. I mean, to me that is a real concern, it determines whether or not I’ll take that shot.”* (focus group 2, male, independent)

Interestingly, when participants were asked how severe a disease had to be in order to accept a vaccine against it, most of the time they mentioned a mental illness, specifically one affecting memory. Apparently they consider physical problems less important and thus less severe. As one woman put it, *“If there would be an injection against Alzheimer’s, I wouldn’t hesitate a moment!”* (focus group 1, female, independent)

One woman also compared the severity of the infectious diseases discussed in the focus group with those that are in the National Immunization Program (NIP). She concluded that it is more important to vaccinate against childhood diseases in light of their severity: *“It also depends on how serious the disease is; for children you can’t take the risk of whooping cough and polio. But for the flu or pneumonia, I think those illnesses are not so serious, well, unless they kill you, of course; normally the inoculation is worse. That is really the decision you have to make. To prevent polio and all those other childhood diseases, you just have to get the shots. Because the risks are too great. But considering the flu and pneumonia, that is not so clear.”* (focus group 1, female, independent)

Others included the number of times one gets the disease in the degree of severity that influences the decision to accept vaccination: *“Well, I think if you get pneumonia once, you don’t immediately drop dead, but if you get it time and again, year after year, then I guess I would be willing to get vaccinated.”* (focus group 1, female, independent)

And last, this perception of the severity of a particular infectious disease can be influenced by personal experience and observing the disease burden on someone else. One woman recounted her husband’s experience of pneumonia: *“Really, my husband had it too. I can tell you, you don’t want to see that happen!”* (focus group 1, female, independent)

Two specific reasons to accept vaccination concern the implications of the severity of the disease. These implications were the probability of contagion, and therefore wanting to protect others, and losing one’s independence.

##### B.3.2. Protection of others

Besides accepting a vaccine to protect oneself against infectious disease, the participants were also willing to protect people around them, especially their grandchildren and vulnerable spouses: *“Especially if you have grandchildren and you know that you are exposing them, then I would always do it, you know.”* (focus group 3, female, residential group) In the same light, people would accept vaccination to prevent becoming sick because of their role in caring for other people. For some, that was the decisive reason to accept the vaccine.

On the other hand, some participants were wary of being tricked into guilt feelings if they rejected the vaccine. *“But we should not be talked into feeling guilty. Because if you don’t participate … .”* (focus group 5, male, residential group) Related to this is the perceived obligation to accept vaccination felt by some of the participants who work with vulnerable people, for example in a nursing home. As one woman explained, *“Well, for me that was the reason to accept a flu shot, because you are always working with older people, who are more vulnerable after all.”* (focus group 11, female, independent) This perceived obligation might be imposed by others: for example, a spouse who needs to be vaccinated in order to babysit for the grandchildren. Participants concluded that vaccination should remain voluntary if the immunization program were to be expanded.

##### B.3.3. Staying independent

Getting an infectious disease could mean losing some independence. The importance of remaining independent was also reflected in the discussion about vaccination. As one participant said, *“No, but you want to remain independent as long as possible, and to me that is a reason to be vaccinated.”* (focus group 11, female, independent) This was especially important for people without a partner. Becoming ill meant that it is more difficult to take care of themselves, and they didn’t want to burden others by asking them to help out. Moreover, they wanted to keep participating and contributing to society. Vaccination could be a means to fulfill this desire: *“It can help you to stay healthy, to be able to participate fully in society.”* (focus group 10, male, independent)

#### B.4 Experiences with previous vaccinations

Another consideration is whether previous vaccination experiences were either positive or negative. Negative experiences with the influenza vaccine led to more hesitation about accepting the next influenza vaccination and perhaps other vaccines as well. One woman told about her experience with the influenza vaccine and its side effects: *“And that is exactly why I often thought that I would not do it again, because it always makes me so sick.”* (focus group 11, female, independent) Experience matters not only with respect to the possible side effects of a vaccine but also to its perceived effectiveness. *“I believe that I will always get my flu shot, because yes, put simply, it works, in my view, I never catch the flu. And when that vaccination was not available, before I got those shots, I did indeed catch the flu once. Clearly, experience counts.”* (focus group 3, female, residential group)

#### B.5 External influence

Two categories of people were identified that influence the decision on whether to accept a vaccination: one’s general practitioner (GP); and one’s friends and family.

##### B.5.1 The general practitioner

The GP plays an important part in the participants’ decision to accept vaccination or not. In the Netherlands, it is currently the GP who invites individuals to come in for an influenza vaccination. Two active roles were identified. The first is leadership, meaning that the GP’s advice is the main reason, and sometimes the only one, to accept or reject vaccination. Having a good relationship with one’s physician and trusting him or her is essential: *“That letter, the invitation to come in for the flu shot, that comes from (…). That is my doctor, and I trust my doctor for 500 %. Period!”* (focus group 3, female, residential group) Even when a participant was initially against vaccination; *“then my doctor convinced me to do it, yes.”* (focus group 13, male, independent). In addition, participants attached great value to the fact that the GP knows their medical condition. They therefore considered their family doctor as the right person to decide whether vaccination is necessary: *“To my mind, he is in the right position to say whether or not it is useful because he knows all your ailments; has the right picture of you.”* (focus group 2, male, independent)

The second active role is an advisory one, meaning that a person will ask the GP for advice, though not necessarily take it. It is added to the rest of the information people gather to make their decision: *“I think it is right, but I also have my own opinion. I would not blindly follow his advice.”* (focus group 7, female, residential group)

Especially the older participants saw the GP as a leader and were more inclined to follow his or her recommendations. The younger participants were less inclined to see the GP as a leader, and they would make their own decision.

And last, some felt that the GP has nothing to do with their decision to accept vaccination: *“I make my own decision, because I form my own opinion of what to do, I make up my own mind, I don’t need the doctor for that.”* (focus group 3, female, residential group)

##### B.5.2 Friends and family

In addition to the GP, other individuals also play a role, though to a lesser extent. These are family and friends. Their role involves talking about vaccination more than giving advice, but especially discussing the experiences, either positive or negative, that influence one’s decision to be vaccinated or not. The conversations can either cast doubt on the effectiveness of the vaccine or reinforce a positive attitude.

### C. Conditions before vaccination is accepted

#### C.1 The need for and influence of different information sources

Besides discussing the various factors that play a role in decision-making, the participants also spoke about the information they would want to receive when a vaccine is offered to them. Obtaining this information is seen as a condition for accepting any vaccination. The participants stressed the need for information about the vaccines: *“Obviously, we are not guinea pigs.”* (focus group 1, female, independent)

Several sources of information were suggested. One idea was to enclose a fact sheet, like the one given with medications, along with the invitation for vaccination. Then the participants would know what to expect, especially regarding the side effects. In addition, other sources of information were discussed, with the GP being the most important one: *“Yes, I would take this up with my doctor, or … Because that would be the right person to provide more information, like how it could turn out, right?”* (focus group 4, female, residential group)

Participants would also search the internet for information and turn to reports on television and in the newspapers. However, these public sources have a different effect on the willingness to accept vaccination and could lead to negative attitudes. Some participants spoke about their mistrust of the medical profession, sparked by reports that money had been earned with the Mexican flu vaccination. Some participants expressed a dislike of the pharmaceutical industry because of this. *“And then there are those publications, on television and in the newspapers, saying that it was all greed, and yes, that made me quite hesitant. I thought, should I go along with it or not? That is obviously not the right thing to do.”* (focus group 5, male, residential group)

As a consequence, the participants wanted a guarantee that information coming from the GP or the government is objective, independent, and research-based. Some participants did express confidence in the government and the medical profession: *“If I could get such a vaccination somewhere, then I would go for it. And if it wouldn’t do any good, they wouldn’t be offering it; that is the only thing I wrote down.”* (focus group 10, female, independent)

## Discussion

We identified eight themes that influence the decision of persons aged 50 years and older to accept vaccination. These are healthy aging, usefulness of vaccination in older age, risk of getting an infectious disease, vaccine characteristics, severity of the disease, experiences of previous vaccinations, influence of healthcare workers and other people, and need for information.

Vulnerability to infectious diseases as experienced by the participants and the usefulness of vaccination in older age seem to be the most important factors influencing the decision to accept a vaccine. Concerning vulnerability, the participants fall into two distinct groups. The first did not feel vulnerable to infectious diseases, often due to their healthy lifestyle. The second did feel vulnerable because they suffer from chronic disease, have themselves already experienced disease previously, or someone near to them had. This is an important finding because the core argument for offering vaccination to older people is their biological susceptibility to infection [2].

With regard to the usefulness of vaccination in older age, it were mostly the older participants who expressed doubts. Questions were raised about vaccines that could prolong life. Life is seen as finite; it should not be prolonged at all costs, especially when death could bring deliverance from suffering. In addition, the participants felt that aging should occur normally without any interventions. They saw more need for vaccination at a younger age, though this would depend on one’s health status at the time of vaccination.

The usefulness of vaccination in older age is scarcely treated in the literature [10]. We feel that this theme came to light because of our explorative design and the fact that the study concerns not only the current offerings but also adding more vaccines to the program. Participants were asked to give all reasons they might have for accepting vaccinations (not only influenza immunization) instead of asking about specific ones. This might have given the participants a cue to take a broader perspective, allowing umbrella arguments to come to mind.

Furthermore, as part of the theme of severity of the disease and its implications, the motive of wanting to protect others emerged in the focus groups as a reason to accept vaccination. This is another topic that has not been addressed in other studies. It is briefly touched upon in Kwong *et al.* [11]. There, the participants believed the vaccine would protect themselves and their family around them, especially the grandchildren. Also in our study, the participants expressed a need for vaccination in order to babysit for their grandchildren and to comply with the wishes of their children, as well as to protect their ill spouses. It is not clear why this topic was pronounced in our study but less so in others. It might reflect the increasing attention given to vaccinating adults in the Netherlands in recent years, after the Health Council released a report on moving toward a vaccination program for all ages [12]. This may have raised awareness of immunization among general practitioners, which in turn could have led to offering more vaccinations or giving more information on the availability of vaccines. The motive of protecting others could be useful when considering vaccination against whooping cough (pertussis), which was regarded as a terrible childhood disease. The participants were often willing to accept pertussis vaccine in order to protect their grandchildren from it.

Overall, in contrast to our findings, (dis)trust in medicine and medical personnel and in the health services in general is often mentioned in the literature, notably in the studies of Telford and Rogers, Harris and Evans [13–15]. Also, in contrast to others who found that logistic problems and/or financial barriers could impede the acceptance of vaccination [16], our study did not identify any logistic problems. This divergence might be related to the fact that in the Netherlands the influenza vaccine is provided by the general practitioner. Logistics are therefore not much of an issue because 75 % of the population have access to their GP within less than a kilometer [17]. Furthermore, influenza vaccine is given free of charge, which could explain why cost is irrelevant to the participants in our study.

The findings of our qualitative study still need to be explored quantitatively. Nonetheless, our results suggest that targeted messages or personalized vaccination could be the key to a high vaccination uptake when offering older adults other vaccines alongside the existing influenza vaccination program. Information should be objective and independent. Information providers should also take into account that younger and older people may have different attitudes on some of the factors that were identified and illustrated in this study. Given that some older adults seem not to prefer prolonging life but would rather pursue quality of life, they might be more motivated to accept herpes zoster vaccination than pneumococcal vaccination. Although others might have the opposite inclination toward these vaccines, our findings indicate that the focus should not be solely on prolonging life.

Second, the participants did not feel vulnerable in general. Every person has a risk of infection at some time in life. However, older adults have an extra risk factor, namely their age. In order to give older adults the opportunity to fully profit from available vaccinations, this risk information could be shared. The ideal person to provide such information would be their GP. He or she has records of disease history and other information on patients in their clinics that would give them the background for a more precisely targeted advice. Moreover, the GP is by definition an important person in the decision-making process of older adults, as shown by our study especially for the elderly.

However, the younger participants preferred to rely on the internet for guidance in their decision on whether to accept vaccination. Special attention is therefore needed to ensure that appropriate information is easy accessible on the internet. Nevertheless, it still has to be recognized that some participants did not consider vaccination useful in older age.

Whereas most studies consider vaccination programs in their current composition, we looked into adding vaccines to existing influenza programs. Examples of potential additions are herpes zoster vaccine, pertussis vaccine, and most notably pneumococcal vaccine. A particular strength of this study is its broad explorative design. No pre-specified models were used to guide the topics that would be raised in the focus groups. This allowed the participants to speak freely about vaccination. That might explain why some themes that had not been covered before came up in the sessions, such as usefulness of vaccination in older age.

There are also a few limitations to this study. Unfortunately, we did not gather demographics on the participants except for their age and residential setting. Had we done so, we would have been able to distinguish between the individual participants and their views on vaccination.

Furthermore, the persons who participated in the focus groups were probably already interested in research, which could imply a selection bias. In addition, the gender distribution was not balanced, with 65 women and 15 men, so the attitudes of men are underexposed.

We tried to recruit a representative sample by inviting persons aged 50 years and older from different residential settings across the Netherlands. Unfortunately, we were only able to recruit two persons living in a care home. We approached several care homes, but the administrators usually refused to cooperate because the residents were not deemed able to participate.

Ideally we would like to have selected the participants randomly. However, this was not feasible because of the focus-group setting. The participants may have had to make arrangements for long travel and we would have needed enough individuals to form the focus groups. Three of the 13 focus groups had less than the minimum of five participants, but the shortfall was due to illnesses on the day they were convened. It is unfortunate that with this recruitment method it is not known how many persons were invited to take part, but we feel this was the most feasible approach.

Last, seven of the 13 focus groups were convened at a time when the media carried frequent reports on the role the pharmaceutical industry allegedly played in the provision of vaccines. This publicity could have influenced the results of our study because, as mentioned earlier, the participants would gather some of their information from the media. Still, mistrust was not found to be a key theme in this study, so the media influence may be considered low.

## Conclusions

In conclusion, this qualitative study shows that the decision to accept vaccination is based not on a single argument but on multiple. The absence of perceived susceptibility seems to be the most important reason to reject vaccination. It is important to realize that the views of persons 50 years and older might be age-related. Furthermore, some of their views on health might relate to their intentions regarding vaccination and their preferences for specific vaccines (*i.e.*, protecting quality of life versus life prolongation). In that light, a targeted or even personalized approach might be the most suitable way to encourage older adults to accept vaccination offers. Moreover, the usefulness of vaccination in older age must be taken into consideration. These observations warrant further exploration in future research.

### Ethics

This type of study does not require ethics approval in the Netherlands because it does not fall under the Medical Research Involving Human Subjects [18].

## Additional file

Additional file 1:
**An overview of the semi-structured questions used during the focus group.**
