# Supplementary material for: Motives of Dutch persons aged 50 years and older to accept vaccination: a qualitative study
Source: BMC Public Health. 2015 May 16;15:493. doi: 10.1186/s12889-015-1825-z (PMC4446004; doi:10.1186/s12889-015-1825-z)
Supplement: Additional file 1: — An overview of the semi-structured questions used during the focus group. [file 12889_2015_1825_MOESM1_ESM.docx]

Additional file 1 An overview of the semi-structured questions used during the focus group

Introductory questions:

- When hearing the concept ‘Healthy Ageing’, what are your thoughts? Are there any specific actions you undertake to obtain Healthy Ageing?
- Is vaccination part of Healthy Ageing?
- What are your thoughts on vaccination in general?

Key questions:

- In general; What reasons do you have to accept vaccination and what reasons do you have to don’t accept vaccination?
- When is an infectious disease severe enough to accept vaccination?
- Do you feel vulnerable for acquiring an infectious disease?
- To which extent would side-effects be acceptable?
- What would be conditions for accepting vaccination? What are situations when you would definitely not would accept vaccination?
- Concerning the effectivity of the vaccine; are there differences between infectious disease? Is there a lower limit?
- Does the general practitioner play a role in your vaccination decision-making? If so, what is that role? What would you do if the GP recommends the vaccine or not recommends it?
- Is the general practitioner trustworthy?
- Are there other persons important to seek advice?

Closing questions:

- Could you rank the different aspects we spoke about regarding your vaccine decision-making?
- Are there any aspects we did not discuss that you would like to mention?
